# Clinical Profile and Outcome of Bronchiolitis in Children With 1-24 Months of Age

**DOI:** 10.7759/cureus.69640

**Published:** 2024-09-18

**Authors:** Sanghavi B, Sugapradha GR, Belgin Premkumar, Joan Elizabeth

**Affiliations:** 1 Paediatrics, Trichy Sri Ramaswamy Memorial (SRM) Medical College Hospital and Research Centre, Trichy, IND

**Keywords:** breastfeeding, low birth weight, passive smoking, preterm, socioeconomic status

## Abstract

Introduction

Bronchiolitis poses a significant challenge in pediatric critical care. It is an acute illness affecting the lower respiratory tract in children under the age of two. The most common cause of bronchiolitis is the seasonal respiratory syncytial virus, with influenza and adenovirus also notable contributors. It is characterized by various clinical symptoms and indicators, such as an upper respiratory prodrome, increased respiratory effort, and wheezing in younger children under two years old. This study primarily examines the clinical profile, risk factors, severity, and outcomes of bronchiolitis in children under two years, excluding the neonatal age group.

Materials and methods

Children under two years of age who presented with upper respiratory symptoms and their first episode of wheezing were evaluated. Those with pre-existing systemic conditions such as cardiac, respiratory, or immunodeficiency disorders were excluded. A detailed history was gathered using a questionnaire, and risk factors were analyzed. The severity of the condition was measured using the Wood-Downes-Ferres score. Data analysis was performed using IBM SPSS Statistics for Windows, Version 26.0 (Released 2019; IBM Corp., Armonk, New York, United States). The relationship between risk factors, severity, and outcomes was examined using the chi-squared test. A two-sided probability of p<0.05 was considered statistically significant for all tests.

Results

Among 54 children aged 1-24 months with bronchiolitis, the average age was 10.18 months, with a standard deviation of 4.8 months. The severity of the condition was greater in younger children (1-12 months) and tended to decrease with age. Bronchiolitis was more common in males (33 cases) than females (21 cases). Approximately 50 children (92.6%) exhibited signs of respiratory distress, and 45 children (83.3%) showed cough as an initial symptom. Severity was notably higher in children with a history of irritability, which was statistically significant (chi-squared value: 8.169; p-value: 0.017). Only 16 children (29.65%) had a history of poor feeding. Bronchiolitis was more prevalent among infants with a birth weight under 1500 grams (63%). Non-exclusive breastfeeding and early bottle feeding were significant risk factors for bronchiolitis and its severity (chi-squared values: 18.794; p-value: 0.000 and 7.795; p-value: 0.020, respectively). Only two children (3.7%) had been exposed to passive smoke, and the severity was slightly higher in these cases. There was also a statistically significant association between socioeconomic status and bronchiolitis (chi-squared value: 11.917; p-value: 0.018).

Conclusion

This study aims to raise awareness among parents and clinicians about the high-risk age group for bronchiolitis, its typical presentations, and predictors of severity. It underscores the impact of both biological and environmental risk factors, such as bottle feeding, non-exclusive breastfeeding, and socioeconomic status, on the severity of the condition.

## Introduction

Bronchiolitis presents a significant challenge in pediatric critical care, affecting the lower respiratory tract in children under two years of age. According to the American Academy of Pediatrics (AAP) guidelines, bronchiolitis is marked by acute inflammation, edema, necrosis of the epithelial cells lining the small airways, and increased mucus production. The condition typically starts with rhinitis and cough, which may progress to lower respiratory tract symptoms such as tachypnea, wheezing, rales, use of accessory muscles, and nasal flaring [1]. Various environmental and biological risk factors, including age, sex, breastfeeding practices, bottle feeding, passive smoke exposure, family type, socioeconomic status, initial symptoms, gestational age, and birth weight, play a significant role in the development of bronchiolitis [2]. Respiratory syncytial virus (RSV) is the most commonly isolated pathogen, found in about 75% of cases (30-70% in Indian studies), while other viruses like rhinovirus, parainfluenza, adenovirus, human metapneumovirus, and bocavirus also contribute to the condition. Mycoplasma is more commonly associated with bronchiolitis in older children [3]. This study aimed to assess the clinical profile, outcomes, severity, and associated risk factors of bronchiolitis in children aged 1-24 months.

## Materials and methods

Study design

This is a prospective descriptive study.

Sample size

This study has 54 participants.

Study population

Children aged 1-24 months experiencing their first episode of wheezing and admitted to the pediatrics department of Trichy Sri Ramaswamy Memorial (SRM) Medical College Hospital and Research Centre in Trichy, India, comprised the study population.

Data collection

Children under two years old presenting with upper respiratory symptoms followed by respiratory distress signs, such as tachypnea and retractions, and their first episode of wheezing, were evaluated. Children with underlying systemic conditions, including cardiac, respiratory, and immunodeficiency disorders, were excluded. A detailed history was collected using a simple questionnaire, assessing risk factors such as birth weight, exclusive breastfeeding, bottle feeding, parental asthma, type of family, socioeconomic status (modified Kuppuswamy scale), and passive smoking. Severity was evaluated using the Wood-Downes-Ferres score (Table 1).

**Table 1:** Wood-Downes-Ferres score Score: 1-3: mild bronchiolitis; 4-7: moderate bronchiolitis; 8-14: severe bronchiolitis

	0	1	2	3
Wheezing	None	End expiration	Entire expiratory phase	Inspiration and expiration
Retractions	None	Subcostal and intercostal	1+supraclavicular+nasal flaring	2+suprasternal+lower intercostal
Respiratory rate (breaths/minute)	<30	31-45	46-60	>60
Heart rate (beats/minute)	<120	>120		
Inspiratory breath sounds	Normal	Regular, symmetrical	Markedly silent, symmetrical	Silent thorax, no wheezing
Cyanosis	Not present	Present		

Inclusion criteria

Children aged 1-24 months with their first episode of wheezing were included in the study.

Exclusion criteria

Children under one month or over 24 months, those with a previous history of wheezing, or those with underlying systemic illness or immunodeficiency conditions were included in the study.

Ethical approval

Approval was obtained from the Institutional Ethical Committee (IEC) of Trichy Sri Ramaswamy Memorial (SRM) Medical College Hospital and Research Centre (approval number: 1136/TSRMMCH&RC/ME-1/2023-IEC No: 170).

## Results

In this study, 54 children with bronchiolitis were analyzed, with a mean age of 10.18 months and a standard deviation of 4.8 months. Among them, 48 children were aged 1-12 months, and six were aged 13-24 months. The severity of bronchiolitis was greater in the younger age group (1-12 months) and decreased with increasing age. However, the difference in severity across age groups was not statistically significant (chi-squared value: 0.213; p-value: 0.899). Bronchiolitis was more prevalent in males (33 cases) compared to females (21 cases), with a slightly higher severity observed in males, although this difference was not statistically significant (chi-squared value: 2.348; p-value: 0.309) (Table 2).

**Table 2:** Severity based on age group and sex

Characteristics	Severity			Total % (n=54)	Chi-squared value (p-value)
	Mild % (n=10)	Moderate % (n=38)	Severe % (n=6)		
Age group					
<1 year	18.8 (9)	70.8 (34)	10.4 (5)	88.9 (48)	0.213 (0.899)
>1 year	16.7 (1)	66.6 (4)	16.7 (1)	11.1 (6)	
Gender					
Male	21.2 (7)	72.7 (24)	6.1 (2)	61.1 (33)	2.348 (0.309)
Female	14.3 (3)	66.7 (14)	19 (4)	38.9 (21)	

Out of the 54 children with bronchiolitis, 50 (92.6%) presented with signs of respiratory distress, such as rapid breathing and retractions, while 45 (83.3%) presented with cough as an initial symptom. Nine children (16.7%) had other viral prodromal symptoms like coryza. Cough, however, was not statistically associated with the severity of illness (chi-squared value: 0.101; p-value: 0.951) (Table 2). A total of 17 children (31.5%) had a history of irritability, while 37 children (68.5%) did not. Increased severity was significantly associated with a history of irritability (chi-squared value: 8.169; p-value: 0.017). Among the children, only 16 (29.65%) had a history of poor feeding, with slightly higher severity noted in these cases, though this was not statistically significant (chi-squared value: 3.111; p-value: 0.211). Prematurity was not found to be a risk factor for bronchiolitis, as there was no significant association with severity (chi-squared value: 1.075; p-value: 0.584). However, bronchiolitis was more common in babies with very low birth weight (<1500 grams) (63%) compared to those with normal birth weight (9.3%) and low birth weight (24.1%). Despite this, the association between birth weight and severity was not statistically significant (chi-squared value: 3.597; p-value: 0.731). Exclusive breastfeeding and bottle feeding were identified as significant risk factors for bronchiolitis and its severity, with both factors showing statistical significance (chi-squared value: 18.794; p-value: 0.000 for exclusive breastfeeding; chi-squared value: 7.795; p-value: 0.020 for bottle feeding). Only 15 children (9.3%) had parents with a history of asthma, and this was not statistically significant in relation to bronchiolitis severity. Most children did not have a history of exposure to passive smoking; only two children (3.7%) had such exposure. While severity was slightly higher in those exposed to passive smoke, this finding was not statistically significant (chi-squared value: 0.874; p-value: 0.646). Family type did not significantly influence bronchiolitis severity; 59.3% of the children lived in nuclear families, with no substantial difference in severity. However, socioeconomic status was significantly associated with the severity of bronchiolitis (chi-squared value: 11.917; p-value: 0.018). Fifty percent (27 children) with severe bronchiolitis were from class III socioeconomic status, followed by 40.7% (22 children) from class II (Table 3).

**Table 3:** Severity based on risk factors

Characteristics	Severity			Total% (n=54)	Chi-squared value (p-value)
	Mild	Moderate	Severe		
Respiratory distress					
Yes	18 (9)	70 (35)	12 (6)	92.6 (50)	0.591 (0.744)
No	25 (1)	75 (3)	0	7.4 (4)	
Cough					
Yes	17.8 (8)	71.1 (32)	11.1 (5)	83.3 (45)	0.101 (0.951)
No	22.2 (2)	66.7 (6)	11.1 (1)	16.7 (9)	
Irritability					
Yes	0	76.5 (13)	23.5 (4)	31.5 (17)	8.169 (0.017)
No	27 (10)	67.6 (25)	5.4 (2)	68.5 (37)	
Poor feed					
Yes	6.3 (1)	75 (12)	18.7 (3)	29.6 (16)	3.111 (0.211)
No	23.7 (9)	68.4 (26)	7.9 (3)	70.4 (38)	
Gestational age					
Preterm	20 (2)	60 (6)	20 (2)	18.5 (10)	1.075 (0.584)
Term	18.2 (8)	72.7 (32)	9.1 (4)	81.5 (44)	
Birth weight					
Normal	20 (1)	80 (4)	0	9.3 (5)	3.597 (0.731)
Low	15.4 (2)	61.5 (8)	23.1 (3)	24.1 (13)	
Very low	20.6 (7)	70.6 (24)	8.8 (3)	63 (34)	
Extremely low	0	100 (2)	0	3.7 (2)	
Exclusive breastfeeding					
Yes	20.5 (9)	77.2 (34)	2.3 (1)	81.5 (44)	18.794 (0.000)
No	10 (1)	40 (4)	50 (5)	18.5 (10)	
Bottle feed					
Yes	16.7 (2)	50 (6)	33.3 (4)	22.2 (12)	7.795 (0.020)
No	19 (8)	76.2 (32)	4.8 (2)	77.8 (42)	
Parental asthma					
Yes	20 (1)	80 (4)	0	9.3 (5)	0.691 (0.708)
No	18.4 (9)	69.4 (34)	12.2 (6)	90.7 (49)	
Type of family					
Nuclear	15.6 (5)	71.9 (23)	12.5 (4)	59.3 (32)	1.038 (0.904)
Joint	22.7 (5)	68.2 (15)	9.1 (2)	40.7 (22)	
Socioeconomic class					
Class 2	13.6 (3)	86.4 (19)	0	40.7 (22)	11.917 (0.018)
Class 3	14.8 (4)	66.7 (18)	18.5 (5)	50 (27)	
Class 4	60 (3)	20 (1)	20 (1)	9.3 (5)	
Passive smoking					
Yes	0	100 (2)	0	3.7 (2)	0.874 (0.646)
No	19.2 (10)	69.3 (36)	11.5 (6)	96.3 (52)	

## Discussion

In developing countries like India, bronchiolitis poses a significant challenge among young infants. Our study indicates that most affected children are under one year of age and severity is notably higher in this younger group. This observation aligns with studies by Hall et al. and Uyan et al., where 85% of children were under 12 months [4,5]. Our study's mean age of 10.18 months is higher than the 6.9 months reported by Uyan et al. but similar to the 11.5 months reported by Iqbal et al. [6]. Male predominance was observed in our study (61.1%), consistent with findings from other regions, where male predominance ranges from 58% to as high as 2-24 males per female [5,7]. This predominance is particularly significant in children under 12 months of age in our study. Iqbal et al. found that 91% of children aged two months to two years had respiratory distress at presentation, which supports our finding of 92.6%. Additionally, 29.6% of children in our study had a history of poor feeding, aligning with Iqbal et al.'s finding of 32%. Erickson et al. concluded that respiratory distress is a consistent clinical feature of bronchiolitis, a finding that matches our results [8]. In our study, respiratory distress, cough, poor feeding, and irritability were correlated with the severity of the illness, with irritability showing statistical significance (p=0.017). While bronchiolitis was more prevalent among term babies in our study, the severity was higher in preterm babies, although this difference was not statistically significant (p=0.584). El Basha et al. [9] found that viral etiology was present in 48.6% of preterm and 67% of term infants with bronchiolitis. Despite RSV being more common in term infants, it tends to cause more severe disease in preterm infants, highlighting the need for targeted prophylaxis and management strategies for high-risk preterm infants. Our study found that low and very low birth weight were significant predictors of bronchiolitis severity, supported by Carroll et al., who demonstrated a significant negative association between birth weight and bronchiolitis hospitalization risk [10]. Non-breastfeeding was identified as a significant risk factor for increased disease severity (p=0.000), consistent with findings from Gomez-Acebo et al. and Toms et al. [11,12]. However, Simoes [2] found limited evidence linking non-breastfeeding with increased severe RSV infection risk, which may be explained by variations in the protective effects of breast milk as noted by Toms et al. Parental asthma did not significantly predict disease severity in our study, aligning with Carbonell-Estrany et al. [13]. However, Simoes reported that maternal asthma was associated with less severe disease. Our study also confirmed that exposure to secondhand smoke increases the risk of severe bronchiolitis, consistent with findings from Chen et al. [14] and Kott et al. [15]. Finally, while our study found that children from nuclear families had more severe bronchiolitis compared to those from joint families, this was contrary to other research. Additionally, lower socioeconomic status was associated with more severe disease (p=0.018), which may be attributed to factors such as overcrowding, poor ventilation, and higher birth order. This underscores the impact of socioeconomic conditions on the severity of bronchiolitis in affected children.

## Conclusions

In our study, bronchiolitis was more prevalent in the 1-12-month age group, with a notable male predominance. The majority of cases presented with mild to moderate severity, although severe bronchiolitis was more common among those aged 1-12 months. Respiratory distress, irritability, and poor feeding were the primary symptoms in this age group and were significantly associated with the severity of the condition. Factors such as low birth weight, non-exclusive breastfeeding, bottle feeding, exposure to passive smoking, living in a nuclear family, and lower socioeconomic status were all significantly linked to increased severity of bronchiolitis.
